# The Association of Radiation Dose-Fractionation and Immunotherapy Use With Overall Survival in Metastatic Melanoma Patients

**DOI:** 10.7759/cureus.8767

**Published:** 2020-06-22

**Authors:** Shang-Jui Wang, Sachin R Jhawar, Zorimar Rivera-Nunez, Ann W Silk, John Byun, Eric Miller, Dukagjin Blakaj, Rahul R Parikh, Joseph Weiner, Sharad Goyal

**Affiliations:** 1 Department of Radiation Oncology, Rutgers Cancer Institute of New Jersey, New Brunswick, USA; 2 Department of Radiation Oncology, Ohio State University Comprehensive Cancer Center-James Cancer Hospital and Solove Research Institute, Columbus, USA; 3 Department of Biostatistics and Epidemiology, Rutgers School of Public Health, Piscataway, USA; 4 Department of Medical Oncology, Dana-Farber Cancer Institute, Boston, USA; 5 Department of Radiation Oncology, George Washington University School of Medicine and Health Sciences, Washington, DC, USA

**Keywords:** radiotherapy (rt), hypofractionated rt, immunotherapy, metastatic melanoma, dose-fractionation

## Abstract

Objective

Metastatic melanoma patients often receive palliative radiotherapy (RT) and immunotherapy (IT). However, the immunological interplay between RT dose-fractionation and IT is uncertain, and the optimal treatment strategy using RT and IT in metastatic melanoma remains unclear. Our main objective was to examine the effect of RT dose-fractionation on overall survival (OS).

Methods

Using the National Cancer Database (NCDB), we classified metastatic melanoma patients who received palliative RT into two dose-fractionation groups - conventionally fractionated RT (CFRT; <5 Gy/fraction) and hypofractionated RT (HFRT: ≥5 Gy/fraction) - with or without IT. Survival analysis was performed using the Cox regression model, Kaplan-Meier method, and propensity-score matching (PSM).

Results

A total of 5,281 metastatic melanoma patients were included, with a median follow-up of 5.9 months. The three-year OS was highest in patients who received HFRT+IT [37.3% (95% CI: 31.1-43.5)] compared to those who received HFRT alone [19.0% (95% CI: 16.2-21.9)], CFRT+IT [17.6 (95%CI: 13.9-21.6)], or CFRT alone [8.6% (95%CI: 7.6-9.7); p<0.0001]. The magnitude of OS benefit with the use of IT was greater in those who received HFRT (18.3%) compared with those who received CFRT (9.0%) (p<0.0001). The addition of IT to HFRT, compared to CFRT, was associated with greater OS benefit in patients treated with RT to the brain and soft tissue/visceral (STV) sites. On PSM analysis, HFRT+IT was associated with improved three-year OS compared to other treatments.

Conclusion

Metastatic melanoma patients who received HFRT+IT was associated with the greatest OS benefit. Our findings warrant further prospective evaluation as to whether higher RT dose-per-fraction improves clinical outcomes in metastatic melanoma patients receiving IT.

## Introduction

Melanoma is the first malignancy in which immunotherapy (IT) has gained widespread use in the metastatic setting. Immune checkpoint inhibitors such as anti-cytotoxic T-lymphocyte antigen 4 (CTLA-4) and anti-programmed cell death-1 (PD-1) antibodies, which are now considered first-line therapies in patients with metastatic melanoma, can reverse the immunosuppressive effects exerted by cancer cells and promote antitumor immunity [1-4]. Despite therapeutic advances with IT, patient outcomes can still be improved as responses tend to be limited to a subset of patients who have preexisting T-cell responses that can be reactivated by immune checkpoint blockade [5].

Nearly half of all patients with metastatic melanoma receive radiotherapy (RT) during their treatment, typically in the setting of oligometastases or for palliation of brain metastases, spinal cord compression, or bleeding tumors. RT has been demonstrated to induce immune-modulation through a variety of mechanisms, including increased presentation of antigens, the release of pro-inflammatory cytokines and molecules, upregulation of death receptors and ligands, and neoantigen formation [6]. These immunogenic effects in turn lead to the activation of adaptive antitumor immunity. Thus, the combination of RT with immune checkpoint blockade is a promising therapeutic strategy and has led to the development of clinical trials assessing this combination.

To date, several prospective clinical trials have demonstrated the feasibility, safety, and efficacy of combining IT with RT [7-11]. Recent patterns-of-care studies have also revealed the increasing use of RT and IT in patients with metastatic melanoma [12,13]. However, there is no study available to assess the effect of RT dose-fractionation and timing on overall survival (OS) outcomes of combination therapy. To address this gap in the existing literature, we used the National Cancer Database (NCDB) data on metastatic melanoma to further study the interaction between IT and RT. The primary endpoint of this study was to assess whether the use of IT with hypofractionated RT (HFRT) or conventionally fractionated RT (CFRT) in patients with metastatic melanoma was associated with OS. We also examined the effect of RT treatment site and IT timing on OS. Our hypothesis was that the use of HFRT improves OS compared to CFRT in metastatic melanoma patients receiving IT.

## Materials and methods

Patient population

The NCDB is a national, hospital-based registry sponsored by the American College of Surgeons Commission on Cancer (CoC) and the American Cancer Society. It collects information on approximately 70% of all new invasive cancers diagnosed in the United States annually [14]. Each year, the NCDB receives reports of over one million cancer cases from around 1,500 hospital-based programs accredited by the CoC [14]. The database comprises demographic information, individual diagnosis, and treatment information such as clinical stage, RT dose and volume, and use of IT [15]. The NCDB undergoes extensive internal quality monitoring and validity reviews annually [16]. While the NCDB does not specify the biological agent used for each patient, IT use may include immune checkpoint inhibitors (anti-CTLA-4, anti-PD-1, or anti-PD-L1), interleukins, and oncolytic virus (talimogene laherparepvec), but not BRAF (i.e., dabrafenib or vemurafenib) or MEK (i.e., trametinib) inhibitors.

**Figure 1:**
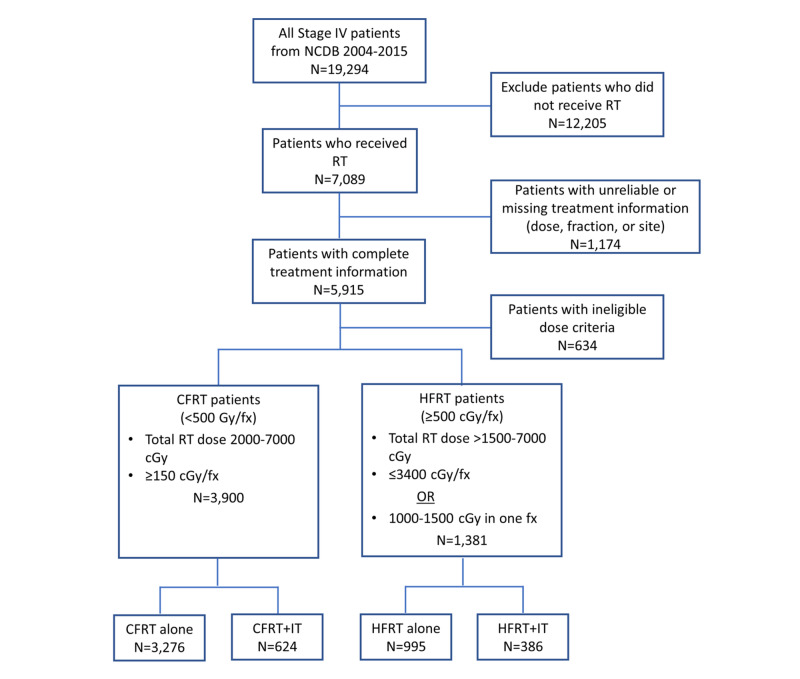
CONSORT diagram detailing patient selection NCDB: National Cancer Database; RT: radiotherapy; CFRT: conventionally fractionated radiotherapy; HFRT: hypofractionated radiotherapy; fx: fraction; IT: immunotherapy

A total of 19,294 patients diagnosed with stage IV melanoma between 2004-2015 were represented in the database. We excluded patients not receiving RT (n=12,205) or with incomplete or unreliable treatment information (e.g., missing total dose, fraction number, or treatment site) (n=1,174). We classified 5,915 patients who received RT with complete treatment information into CFRT (<5 Gy/fraction) and HFRT (≥5 Gy/fraction) dose-fractionation groups (Figure 1). For CFRT, we included 3,900 patients who received total doses of RT between 20-70 Gy and ≥1.5 Gy/fraction. For HFRT, we included 1,381 patients who received total doses of RT between 15.01-70 Gy and ≤34 Gy/fraction, or 10-15 Gy in one fraction. We excluded 634 patients who did not meet the above dose criteria. CFRT and HFRT patients were further divided based on whether they had received IT or not, ultimately resulting in four treatment groups in all (CFRT+IT, CFRT alone, HFRT+IT, and HFRT alone).

**Figure 2:**
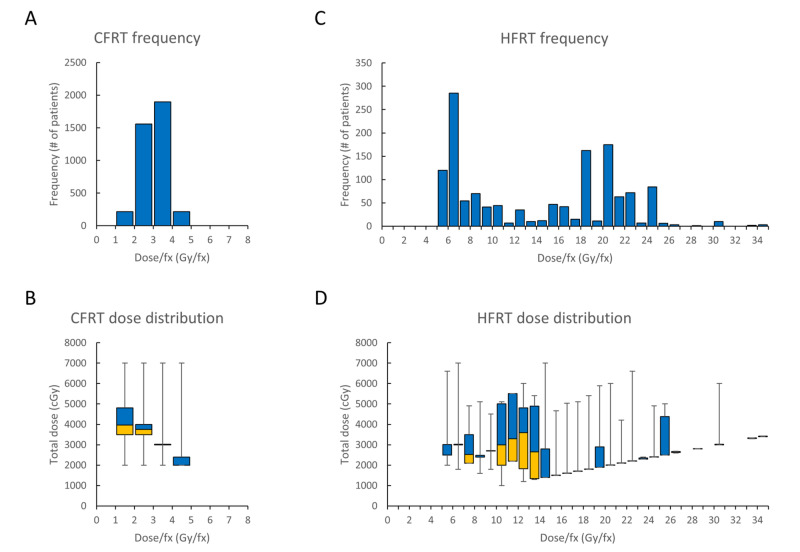
Radiotherapy characteristics of the patient cohort Histograms depict the frequency of patients who received CFRT (A) and HFRT (C) at various dose-per-fraction intervals. Range and distribution of total RT dose are depicted using whisker-box plots for each dose-per-fraction interval in patients who received CFRT (B) and HFRT (D). Dose-per-fraction intervals on the x-axis are inclusive of the smaller value, but not the larger value [e.g., in (A), 1,558 CFRT patients received RT between 2.00-2.99 Gy/fraction]. A whisker-box plot consists of a lower box (yellow, 1st quartile – median), an upper box (blue, median – 3rd quartile), and whiskers (minimum and maximum) CFRT: conventionally fractionated radiotherapy; HFRT: hypofractionated radiotherapy; fx: fraction

Most patients who received CFRT were treated at 2-3 Gy/fraction, with a median dose of 30 Gy [interquartile range (IQR): 7.50], and the frequency and total dose distribution at each dose-per-fraction interval are shown in Figure 2A, 2B. For patients who received HFRT, the median dose was 24 Gy (IQR: 10), and the frequency and total dose distribution are shown in Figure 2C, 2D.

Statistical analysis

Frequencies and proportions were calculated for all demographic and clinical categorical variables. Chi-square and Kruskal-Wallis tests were used to compare proportions and medians. IQRs were calculated to describe median dispersion. Statistical significance was determined at an alpha level of 0.05. Kaplan-Meier survival curves and log-rank statistics were used to examine OS. Survival estimates and 95% CIs were calculated from survival functions at three years. Survival was calculated from the date of diagnosis to the date of the last contact or confirmed death. Cox proportional hazards regression was used to examine the unadjusted hazard ratio (HR) and adjusted hazard ratios (aHR).

Patient variables included in the multivariable Cox regression model were age, sex, Charlson comorbidity score (CCS), education attainment, median household income, type of health insurance, hospital type, chemotherapy use, any type of surgery, and RT treatment sites [brain, bone, and soft tissue/visceral (STV)].

In a sensitivity analysis, we used one-to-one propensity-score matching (PSM) by modeling individual logistic regressions using treatment groups as dependent variable [HFRT-IT group vs. each of the other three treatment groups (HFRT alone, CFRT-IT, and CFRT alone)] with independent variables including age, sex, race, CCS, type of health insurance, and RT treatment site. Cox proportional hazards regression and Kaplan-Meier survival curves were used to examine survival between matched groups. All statistical analyses were performed using SAS software version 9.4 (SAS Institute, Cary, NC).

## Results

Descriptive statistics

Our analysis examined 5,281 patients with stage IV melanoma who met the inclusion criteria (Figure 1). Of those, 7.3% of patients received HFRT+IT, 18.8% received HFRT alone, 11.8% received CFRT+IT, and 62.0% received CFRT alone. The mean age was 62.1 years (range: 19-90 years). Overall, most of the study population was Caucasian (97.0%), had no comorbidities (CCS: 0) (77.9%), with a higher proportion of males (69.9%), and relatively equal distribution of treatments across age groups (Table 1). Patients in the HFRT+IT group had slightly fewer comorbidities (CCS: >0) (p=0.0014) and were more likely to be treated at academic centers (p<0.0001) and to have private insurance (p<0.0001) than those in other treatment groups. Patients who did not receive IT were more likely to receive chemotherapy compared to those who received IT (HFRT/CFRT alone: 25.3% vs HFRT/CFRT+IT: 10.0%; p<0.0001).

**Table 1:** Demographic and clinical characteristics of stage IV melanoma patients (n=5,281) in the NCDB 2004-2015 †Proportion of the number of adults in the patient's zip code who did not graduate from high school RT: radiotherapy; IT: immunotherapy; HFRT: hypofractionated radiotherapy; CFRT: conventionally fractionated radiotherapy; CCS: Charlson/Deyo comorbidity score; STV: soft tissue/visceral; NCDB: National Cancer Database

Patient characteristics	HFRT+IT (N=386), n (%)	CFRT+IT (N=624), n (%)	HFRT (N=995), n (%)	CFRT (N=3,276), n (%)
Age (years)				
≤50	85 (22)	157 (25)	195 (20)	676 (21)
>50-60	106 (27)	159 (25)	217 (22)	770 (24)
>60-70	93 (24)	173 (28)	261 (26)	794 (24)
>70	102 (26)	135 (22)	322 (32)	1036 (32)
Sex				
Female	119 (31)	201 (32)	279 (28)	988 (30)
Male	267 (69)	423 (68)	716 (72)	2288 (70)
Race				
White	377 (98)	608 (98)	957 (96)	3182 (97)
Black	3 (0.8)	7 (1)	12 (1)	48 (1)
Native American	0 (0)	2 (0.3)	1 (0.1)	3 (0.1)
Asian	1 (0.3)	0 (0)	8 (0.8)	16 (0.5)
Other	2 (0.5)	5 (0.8)	6 (0.6)	10 (0.3)
Hispanic ethnicity				
No	370 (96)	593 (95)	931 (94)	3030 (92)
Yes	9 (2)	15 (2)	18 (2)	71 (2)
CCS				
0	320 (83)	507 (81)	798 (80)	2491 (76)
1	51 (13)	77 (12)	132 (13)	534 (16)
2	15 (4)	40 (6)	65 (7)	251 (8)
Income 2008-12, ($)				
≥62,000	167 (43)	230 (37)	359 (36)	1000 (31)
≥47,999-62,999	119 (31)	195 (31)	261 (26)	923 (28)
≥38,000-47,999	73 (19)	119 (19)	219 (22)	818 (25)
<38,000	25 (6)	75 (12)	140 (14)	465 (14)
Education attainment^†^				
<7%	138 (36)	190 (30)	294 (30)	789 (24)
7-12.9%	145 (38)	204 (33)	318 (32)	1126 (34)
13-20.9%	74 (19)	163 (26)	244 (25)	855 (26)
>21%	27 (7)	62 (10)	125 (13)	438 (13)
Insurance				
Private insurance	208 (54)	308 (49)	427 (43)	1263 (39)
Not insured	13 (3)	33 (5)	36 (4)	188 (6)
Medicaid	20 (5)	47 (8)	70 (7)	331 (10)
Medicare	139 (36)	209 (33)	422 (42)	1378 (42)
Other government	3 (0.8)	18 (3)	21 (2)	71 (2)
Hospital type				
Academic/research cancer program	199 (52)	236 (38)	470 (47)	1029 (31)
Community cancer program	7 (2)	46 (7)	28 (3)	302 (9)
Comprehensive community cancer program	95 (25)	232 (37)	283 (28)	1405 (43)
Integrated network cancer program	49 (13)	55 (9)	152 (15)	325 (10)
Chemotherapy				
No	351 (91)	556 (89)	722 (72)	2448 (75)
Yes	35 (9)	66 (11)	268 (27)	814 (25)
Any type of surgery				
No	303 (78)	508 (81)	778 (78)	2678 (82)
Yes	83 (22)	116 (19)	216 (22)	592 (18)
RT treatment site				
Brain	273 (71)	323 (52)	689 (69)	2102 (64)
Bone	33 (9)	119 (19)	62 (6)	467 (14)
STV sites	80 (21)	182 (29)	244 (25)	707 (22)

Survival analysis

In univariate models, most patient characteristics were significantly associated with the risk of dying, except for race, income, and chemotherapy use (Table 2). On multivariate analysis, age of >60 years (aHR range: 1.20-1.60), male sex (aHR: 1.11, 95% CI: 1.03-1.20; p=0.0061), and CCS of ≥1 (aHR range: 1.19-1.22) were associated with increased risk of death. Patients who were not insured (aHR: 1.28, 95% CI: 1.09-1.51; p=0.0029) or on Medicaid (aHR: 1.32, 95% CI: 1.16-1.51; p<0.0001) had higher risk of dying compared to patients on private insurance. However, the most significant factor associated with the risk of dying on multivariate analysis was the type of treatment received. Patients who received CFRT alone (aHR: 2.81, 95% CI: 2.36-3.34; p<0.0001), CFRT+IT (aHR: 1.95, 95% CI: 1.59-2.39; p<0.0001), and HFRT alone (HR: 1.57, 95% CI: 1.31-1.89; p<0.0001) were all at higher risk of dying compared to patients treated with HFRT+IT.

**Table 2:** Univariate and multivariate Cox regression analysis of individual demographic and clinical characteristics with overall survival in the population receiving RT (n=5,281) †Proportion of the number of adults in the patient's zip code who did not graduate from high school RT: radiotherapy; IT: immunotherapy; HFRT: hypofractionated radiotherapy; CFRT: conventionally fractionated radiotherapy; CCS: Charlson/Deyo comorbidity score; STV: soft tissue/visceral; REF: reference; HR: hazard ratio; CI: confidence interval

Patient characteristics	Univariate HR (95% CI)	P-value	Multivariate HR (95% CI)	P-value
Type of treatment				
HFRT+IT	REF		REF	
CFRT+IT	1.73 (1.44-2.09)	<0.0001	1.95 (1.59-2.39)	<0.0001
HFRT alone	1.68 (1.42-2.00)	<0.0001	1.57 (1.31-1.89)	<0.0001
CFRT alone	2.87 (2.45-3.36)	<0.0001	2.81 (2.36-3.34)	<0.0001
Age (years)				
≤50	REF		REF	
>50-60	1.09 (0.99-1.20)	0.0824	1.08 (0.96-1.20)	0.2000
>60-70	1.19 (1.09-1.31)	0.0002	1.20 (1.06-1.35)	0.0035
>70	1.53 (1.40-1.67)	<0.0001	1.60 (1.40-1.82)	<0.0001
Sex				
Female	REF			
Male	1.15 (1.08-1.24)	<0.0001	1.11 (1.03-1.20)	0.0061
Race				
White	REF		REF	
Black	1.02 (0.78-1.34)	0.8761	-	-
Native American	0.96 (0.40-2.30)	0.9238	-	-
Asian	0.69 (0.40-1.19)	0.1792	-	-
Other	0.93 (0.58-1.50)	0.7635	-	-
Hispanic ethnicity				
No	REF		REF	
Yes	0.76 (0.61-0.95)	0.0168	0.84 (0.65-1.08)	0.1737
CCS				
0	REF		REF	
1	1.24 (1.13-1.35)	<0.0001	1.19 (1.08-1.30)	0.0003
2	1.43 (1.27-1.62)	<0.0001	1.22 (1.07-1.39)	0.0036
Income 2008-12, ($)				
≥62,000	REF		REF	
≥47,999-62,999	1.05 (0.97-1.14)	0.2498	-	-
≥38,000-47,999	1.05 (0.96-1.14)	0.2775	-	-
<38,000	1.07 (0.96-1.18)	0.2223	-	-
Education attainment^†^				
<7%	REF		REF	
7-12.9%	1.10 (1.01-1.19)	0.0244	1.03 (0.92-1.16)	0.6182
13-20.9%	1.01 (0.93-1.11)	0.7887	0.94 (0.86-1.04)	0.2104
>21%	1.07 (0.96-1.19)	0.2008	1.07 (0.98-1.17)	0.1347
Insurance				
Private insurance	REF		REF	
Not insured	1.25 (1.09-1.45)	0.0021	1.28 (1.09-1.51)	0.0029
Medicaid	1.27 (1.13-1.42)	<0.0001	1.32 (1.16-1.51)	<0.0001
Medicare	1.38 (1.29-1.48)	<0.0001	1.07 (0.97-1.18)	0.1950
Other government	1.04 (0.83-1.30)	0.7265	0.88 (0.68-1.12)	0.2935
Hospital type				
Academic/research cancer program	REF		REF	
Community cancer program	1.50 (1.33-1.70)	<0.0001	1.24 (1.08-1.42)	0.0018
Comprehensive community cancer program	1.33 (1.23-1.43)	<0.0001	1.16 (1.07-1.25)	0.0003
Integrated network cancer program	1.21 (1.09-1.35)	0.0004	1.14 (1.02-1.28)	0.0254
Chemotherapy				
No	REF		REF	
Yes	1.02 (0.95-1.10)	0.5776	-	-
Any type of surgery				
No	REF		REF	
Yes	0.72 (0.66-0.78)	<0.0001	0.82 (0.75-0.89)	<0.0001
RT treatment site				
STV sites	REF		REF	
Brain	1.58 (1.46-1.71)	<0.0001	1.75 (1.60-1.91)	<0.0001
Bone	1.57 (1.41-1.76)	<0.0001	1.52 (1.35-1.72)	<0.0001

**Figure 3:**
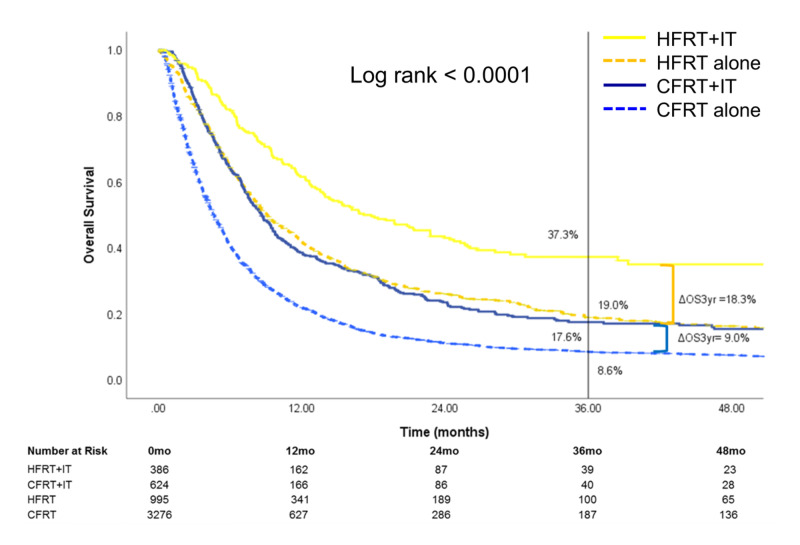
Kaplan-Meier curves for overall survival for entire cohort The entire cohort was stratified by treatment received HFRT: hypofractionated radiotherapy; CFRT: conventionally fractionated radiotherapy; IT: immunotherapy; OS: overall survival

The median follow-up time for the cohort was 5.9 (IQR: 11.4) months. Overall, HFRT+IT patients had the highest three-year OS [37.3% (95% CI: 31.1-43.5)] compared to HFRT alone [19.0% (95% CI: 16.2-21.9)], CFRT+IT [17.6% (95% CI: 13.9-21.6)], and CFRT alone [8.6% (95% CI: 7.6-9.7), overall log rank: <0.0001] (Figure 3). HFRT+IT group had a significantly higher three-year OS compared to the CFRT+IT group (p<0.0001). Compared to RT-alone patients, the addition of IT was associated with improved three-year OS in both HFRT (HFRT alone vs. HFRT+IT, p<0.0001) and CFRT (CFRT alone vs. CFRT+IT, p<0.0001) treatment groups. However, the magnitude of OS benefit associated with IT was significantly larger in the HFRT group (three-year ΔOSIT=18.3%) compared to the CFRT group (three-year ΔOSIT=9.0%, p<0.0001) (Figure 3).

**Figure 4:**
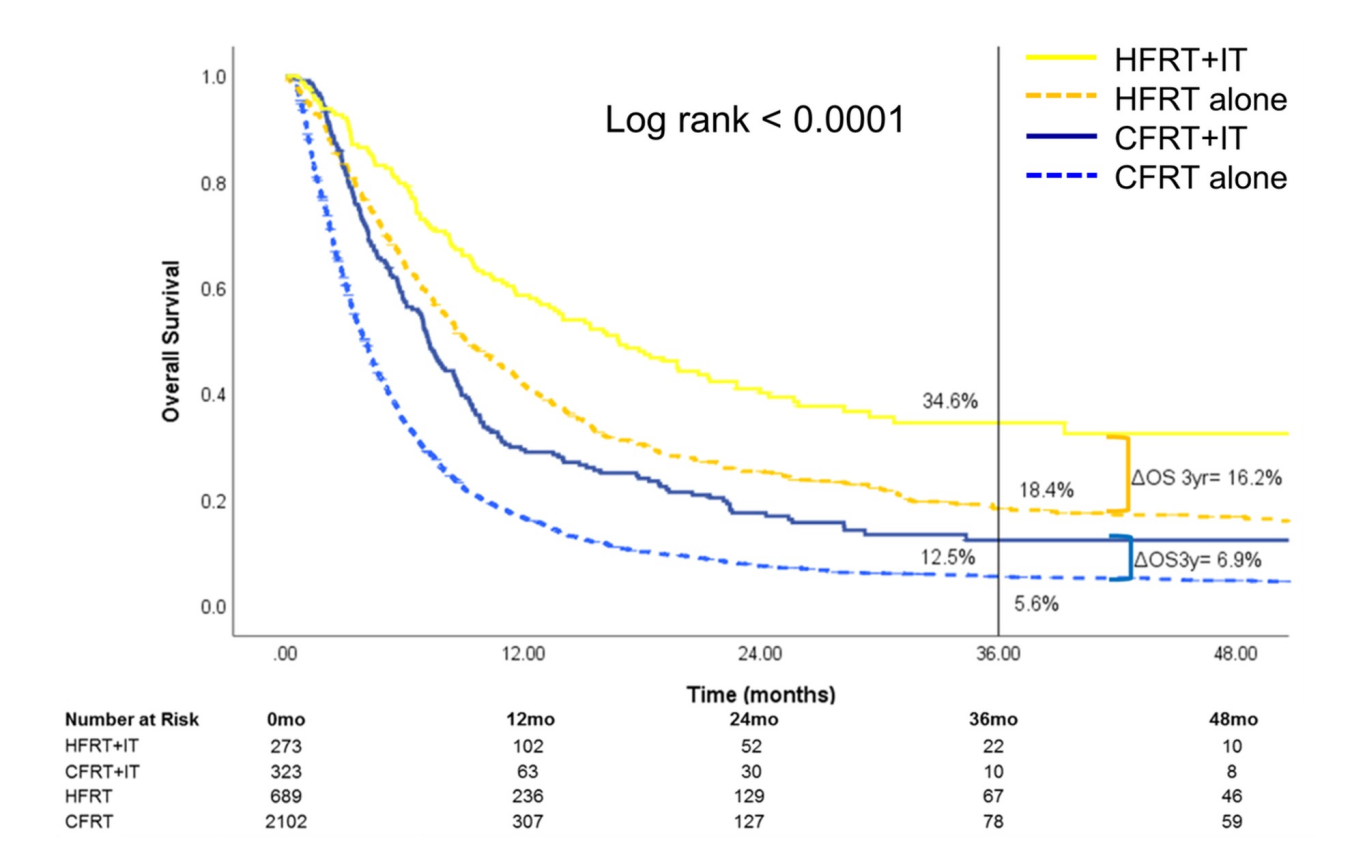
Overall survival of patients who received RT to the brain The subset of patients who received radiotherapy to the brain was stratified by treatment received HFRT: hypofractionated radiotherapy; CFRT: conventionally fractionated radiotherapy; IT: immunotherapy; OS: overall survival

**Figure 5:**
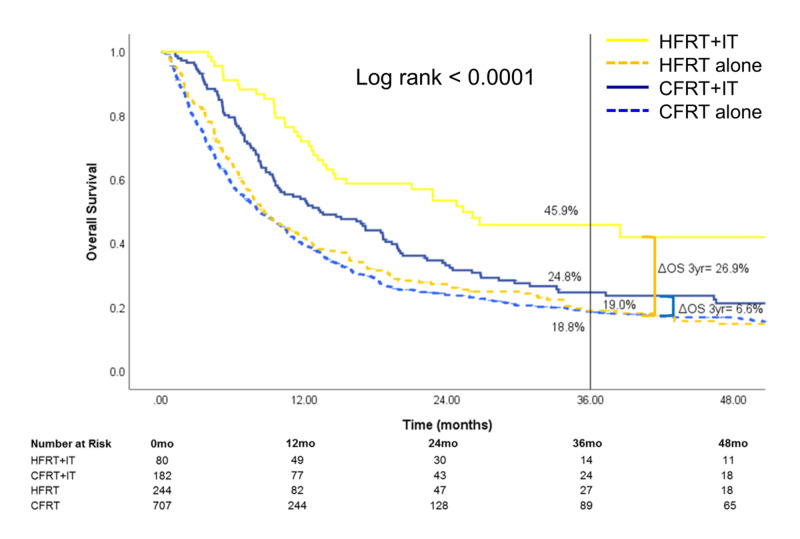
Overall survival of patients who received RT to STV sites The subset of patients who received radiotherapy to STV sites was stratified by treatment received HFRT: hypofractionated radiotherapy; CFRT: conventionally fractionated radiotherapy; IT: immunotherapy; STV: soft tissue/visceral; OS: overall survival

**Figure 6:**
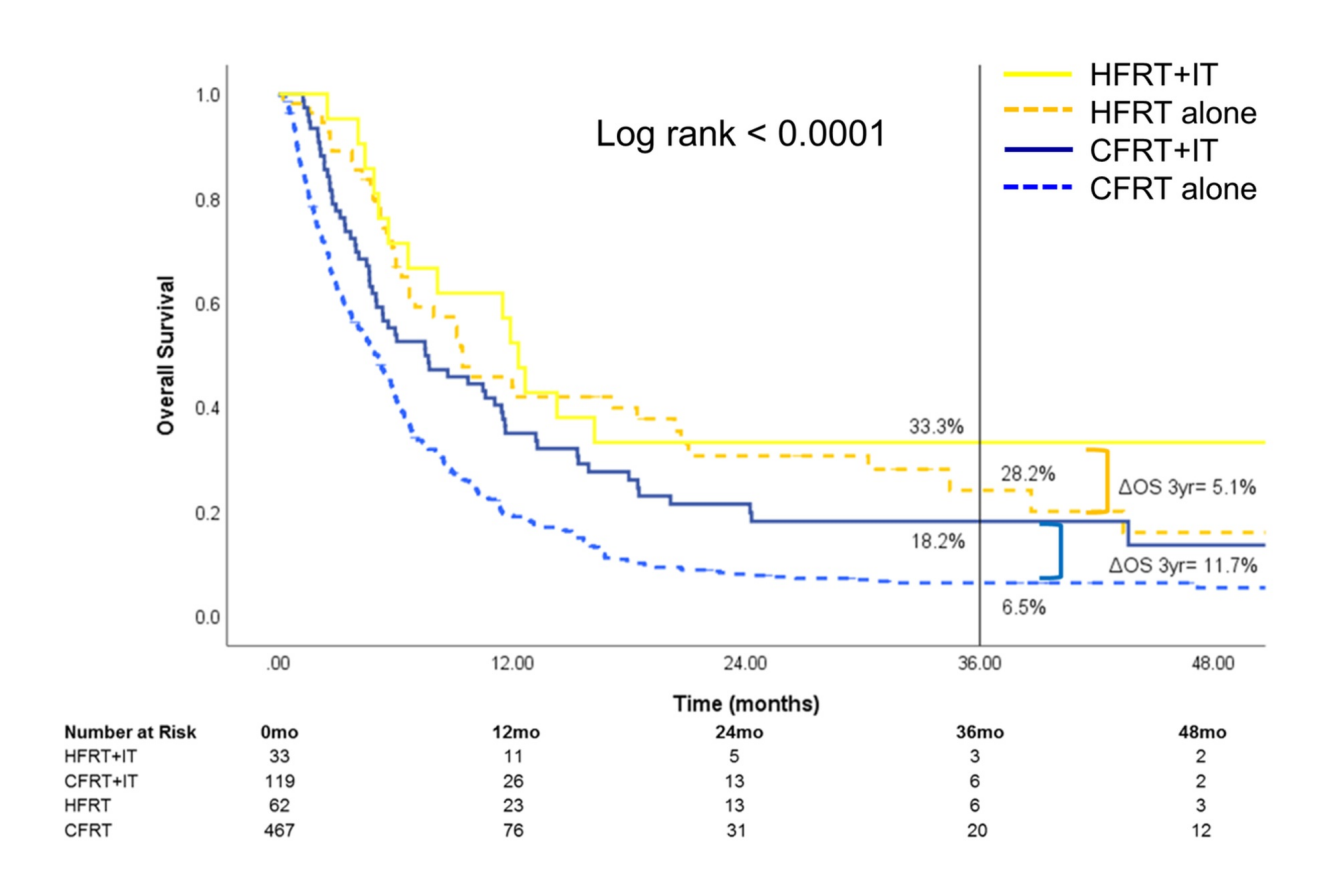
Overall survival of patients who received RT to the bone The subset of patients who received radiotherapy to the bone was stratified by treatment received HFRT: hypofractionated radiotherapy; CFRT: conventionally fractionated radiotherapy; IT: immunotherapy; OS: overall survival

Stratification by RT treatment site (Figures 4-6) showed that the survival benefit of HFRT+IT was evident for brain and STV metastatic sites. For patients treated for metastatic brain sites, three-year OS was highest for HFRT+IT [34.6% (95% CI: 27.1-42.3)] compared to HFRT alone [18.4% (95% CI: 15.2-21.9)], CFRT+IT [12.5% (95% CI: 8.0-18.0)], or CFRT alone [5.6% (95% CI: 4.6-6.8)] (Figure 4). For those treated for metastatic STV sites, three-year OS was also highest for HFRT+IT [45.9% (95% CI: 33.1-57.8)] compared to HFRT alone [19.0% (95% CI: 13.7-25.1)], CFRT+IT [24.8% (95% CI: 17.8-32.4)], or CFRT alone [18.8% (95% CI: 15.7-22.1)] (Figure 5). The OS benefit with the addition of IT was significantly greater in the HFRT group compared to the CFRT group for patients who received RT to the brain (three-year ΔOSIT: HFRT vs CFRT, 16.2% vs 6.9%, p<0.0001) or STV sites (three-year ΔOSIT: HFRT vs CFRT, 26.9% vs 6.0%, p<0.0001) sites. On the other hand, while treatment with HFRT+IT in patients who received RT to bone sites was associated with the highest OS, the addition of IT to HFRT did not significantly improve survival (Figure 6).

**Figure 7:**
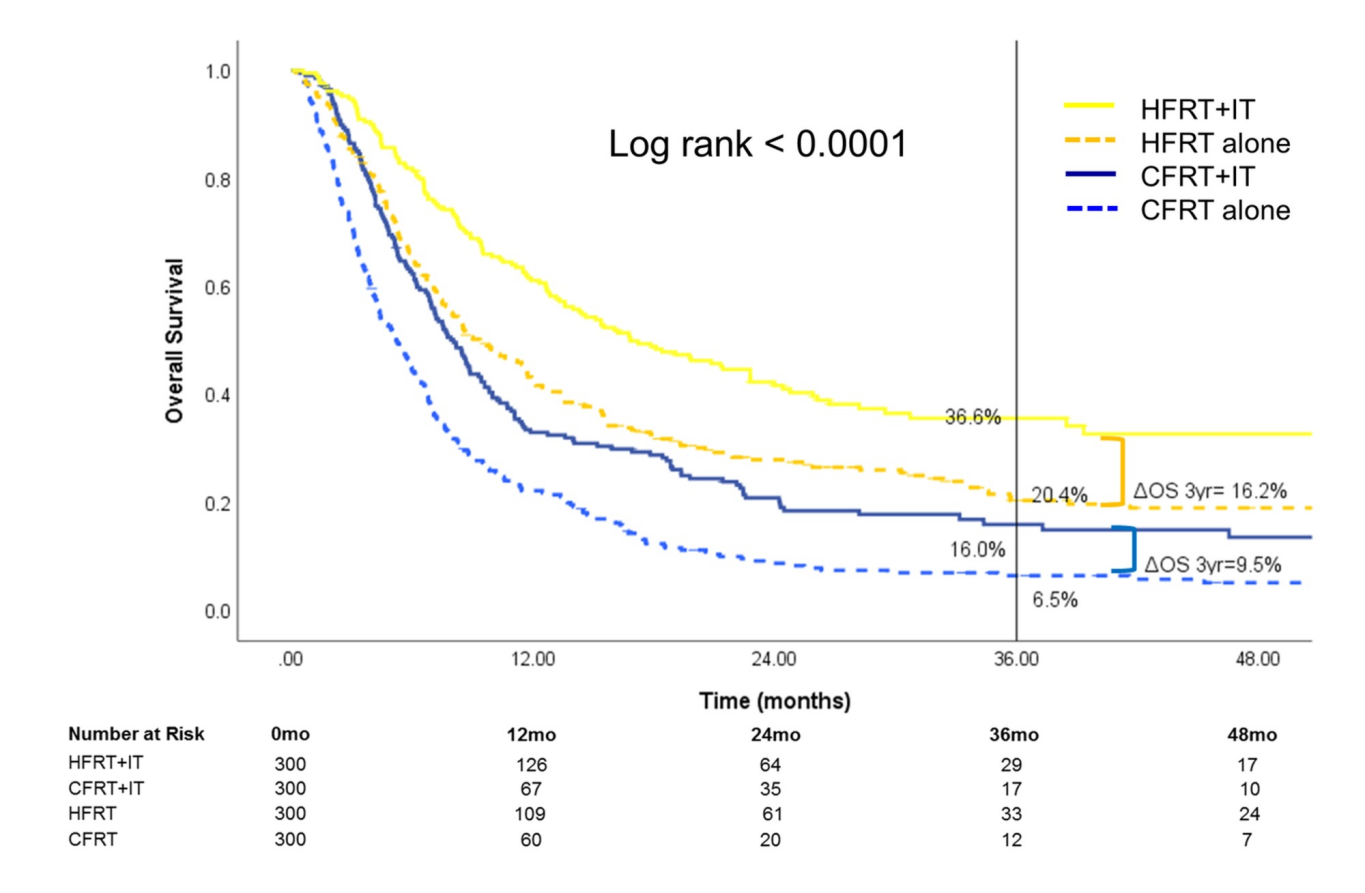
Overall survival after propensity-score matching of 1,200 patients (n=300 for each treatment group) HFRT: hypofractionated radiotherapy; CFRT: conventionally fractionated radiotherapy; IT: immunotherapy; OS: overall survival

After PSM, 1,200 patients (n=300 per treatment group) were available for analysis (Table 3). Treatment with HFRT+IT continued to be associated with superior three-year OS [36.6% (95% CI: 30.0-43.5)] compared to HFRT alone [20.4% (95% CI: 15.4-25.9)], CFRT+IT [16.0% (95% CI: 10.9-21.9)], and CFRT alone [6.5% (95% CI: 3.9-10.0)]. OS benefit associated with the utility of IT remained significantly greater in the HFRT group (three-year ΔOSIT=16.2%) compared with the CFRT group (three-year ΔOSIT=9.5%, p<0.0001) (Figure 7 and Table 4).

**Table 3:** Propensity-score-matched population for stage IV melanoma patients (n=1,200) in the NCDB 2004-2015 RT: radiotherapy; IT: immunotherapy; HFRT: hypofractionated radiotherapy; CFRT: conventionally fractionated radiotherapy; CCS: Charlson/Deyo comorbidity score; STV: soft tissue/visceral; NCDB: National Cancer Database

Patient characteristics	HFRT+IT, n (%)	CFRT+IT, n (%)	HFRT alone, n (%)	CFRT alone, n (%)
Age (years)				
≤50	70 (23)	70 (23)	70 (23)	68 (23)
>50-60	82 (27)	82 (27)	82 (27)	84 (27)
>60-70	76 (25)	76 (25)	76 (25)	76 (25)
>70	72 (24)	72 (24)	72 (24)	72 (24)
Sex				
Female	84 (28)	84 (28)	84 (28)	81 (27)
Male	216 (72)	216 (72)	216 (72)	219 (73)
Race				
White	299 (100)	299 (100)	299 (100)	299 (100)
Others	1 (0)	1 (0)	1 (0)	1 (0)
CCS				
0	10 (3)	10 (3)	10 (3)	10 (3)
1	29 (10)	29 (10)	29 (10)	29 (10)
2	261 (87)	261 (87)	261 (87)	261 (87)
Insurance				
Private insurance	176 (59)	176 (59)	176 (59)	176 (59)
Not insured	5 (2)	5 (2)	5 (2)	5 (2)
Medicaid	10 (3)	10 (3)	10 (3)	10 (3)
Medicare	108 (36)	108 (36)	108 (36)	108 (36)
Other government	1 (0.3)	1 (0.3)	1 (0.3)	1 (0.3)
Site treated				
Brain	218 (72)	218 (72)	218 (72)	218 (72)
Bone	24 (8)	24 (8)	24 (8)	24 (8)
STV sites	58 (19)	58 (19)	58 (19)	58 (19)

**Table 4:** Univariate Cox regression analysis for propensity-score-matched population RT: radiotherapy; IT: immunotherapy; HFRT: hypofractionated radiotherapy; CFRT: conventionally fractionated radiotherapy; REF: reference; HR: hazard ratio; CI: confidence interval

Patient characteristics	Univariate HR (95% CI)	P-value
Type of treatment		
HFRT+IT	REF	
CFRT+IT	1.86 (1.48-2.33)	<0.01
HFRT alone	1.57 (1.26-1.96)	<0.01
CFRT alone	2.96 (2.39-3.66)	<0.01

**Figure 8:**
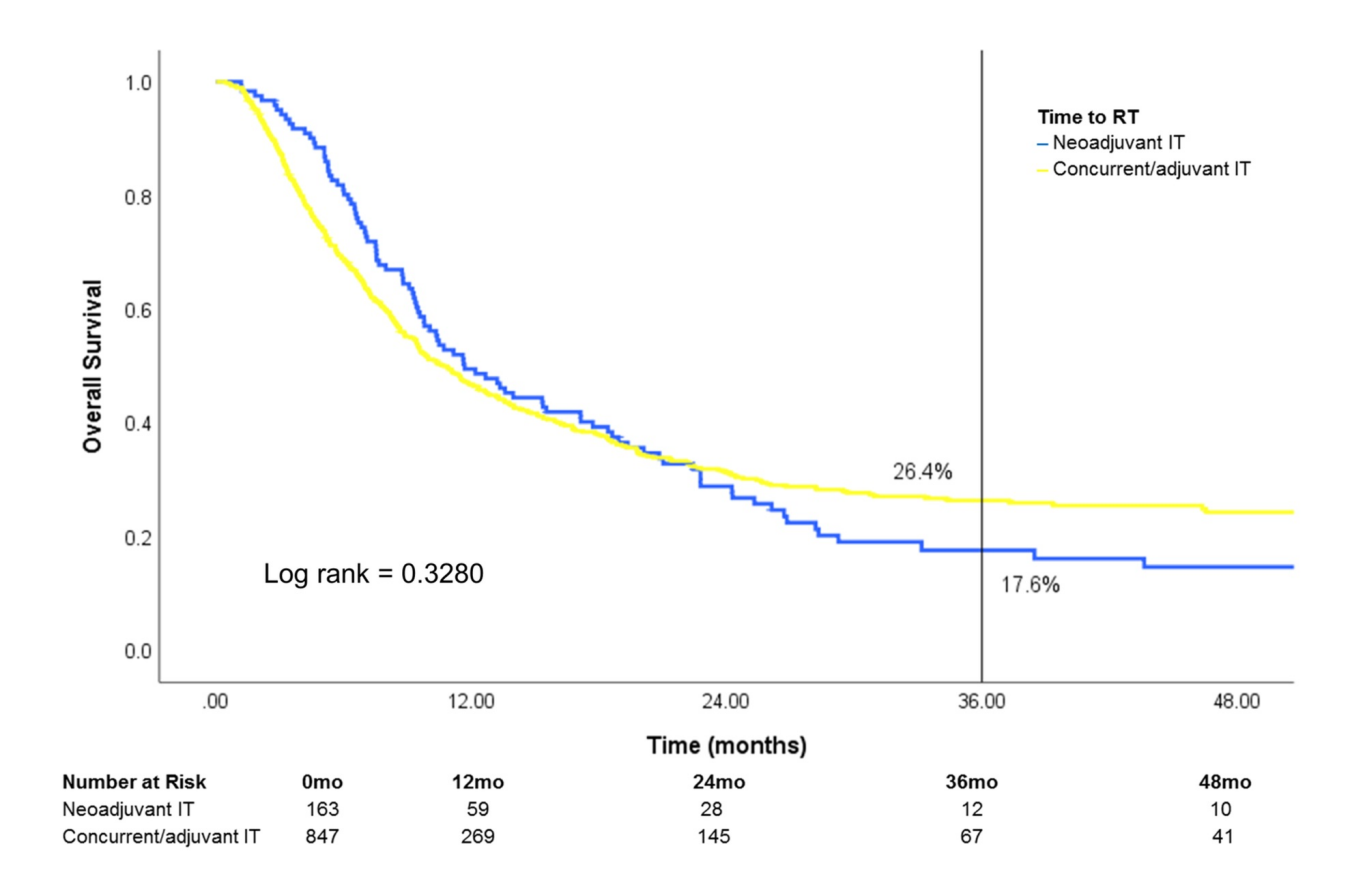
Subset analysis of IT timing on overall survival Patients who received both RT and IT (n=1,010) were stratified by the timing of initiating IT relative to RT RT: radiotherapy; IT: immunotherapy

**Figure 9:**
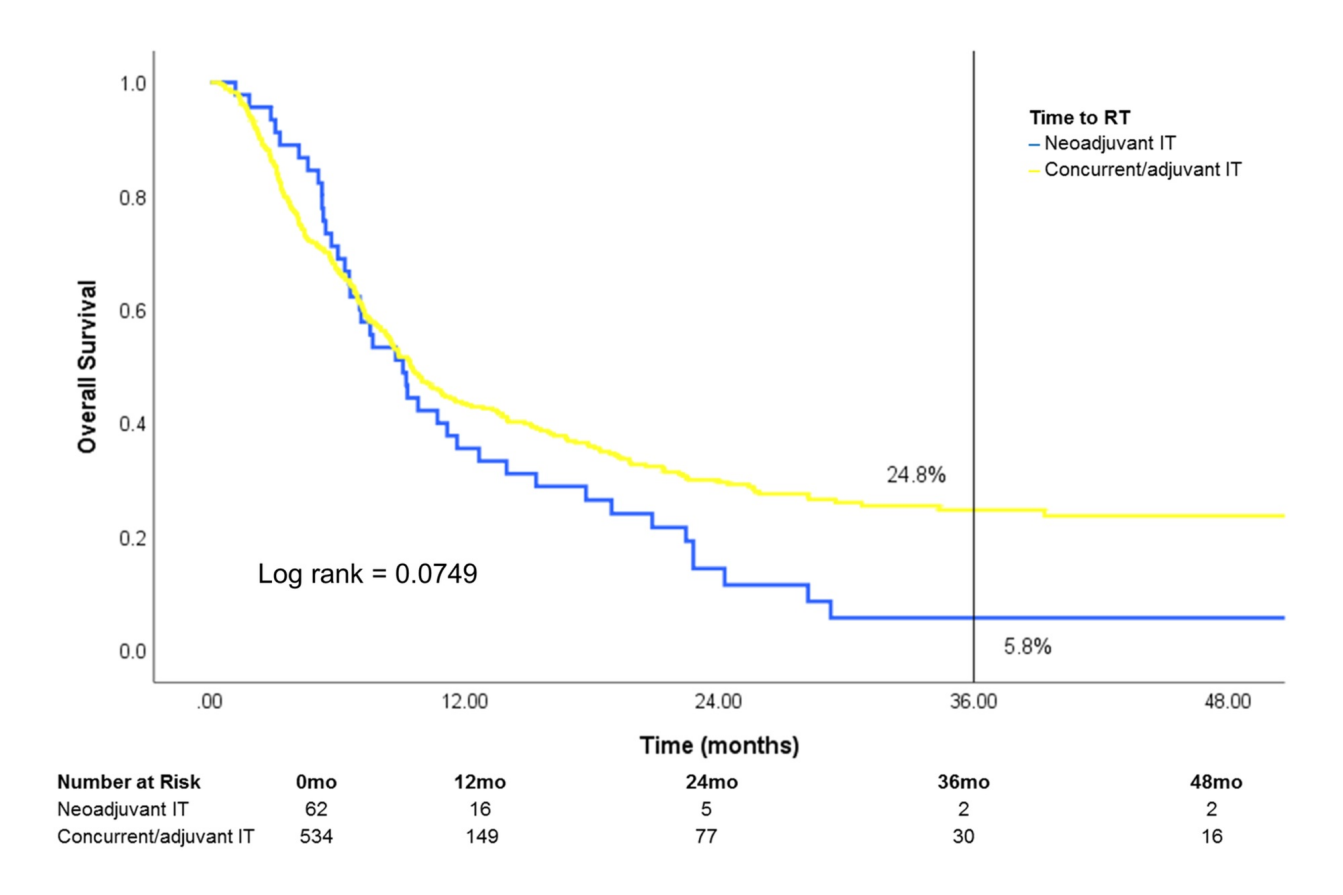
Effect of IT timing on overall survival in patients who received RT to the brain Patients who received RT to the brain and IT (n=596) were stratified by IT timing RT: radiotherapy; IT: immunotherapy

**Figure 10:**
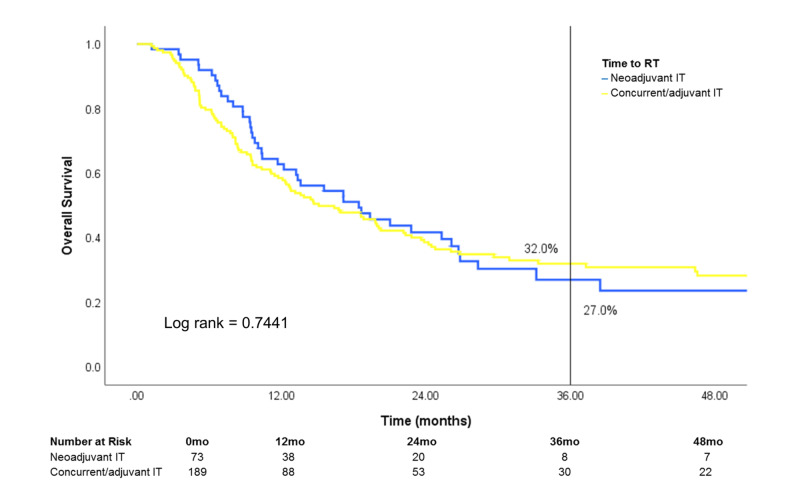
Effect of IT timing on overall survival in patients who received RT to STV sites Patients who received RT to STV sites and IT (n=262) were stratified by IT timing RT: radiotherapy; IT: immunotherapy; STV: soft tissue/visceral

**Figure 11:**
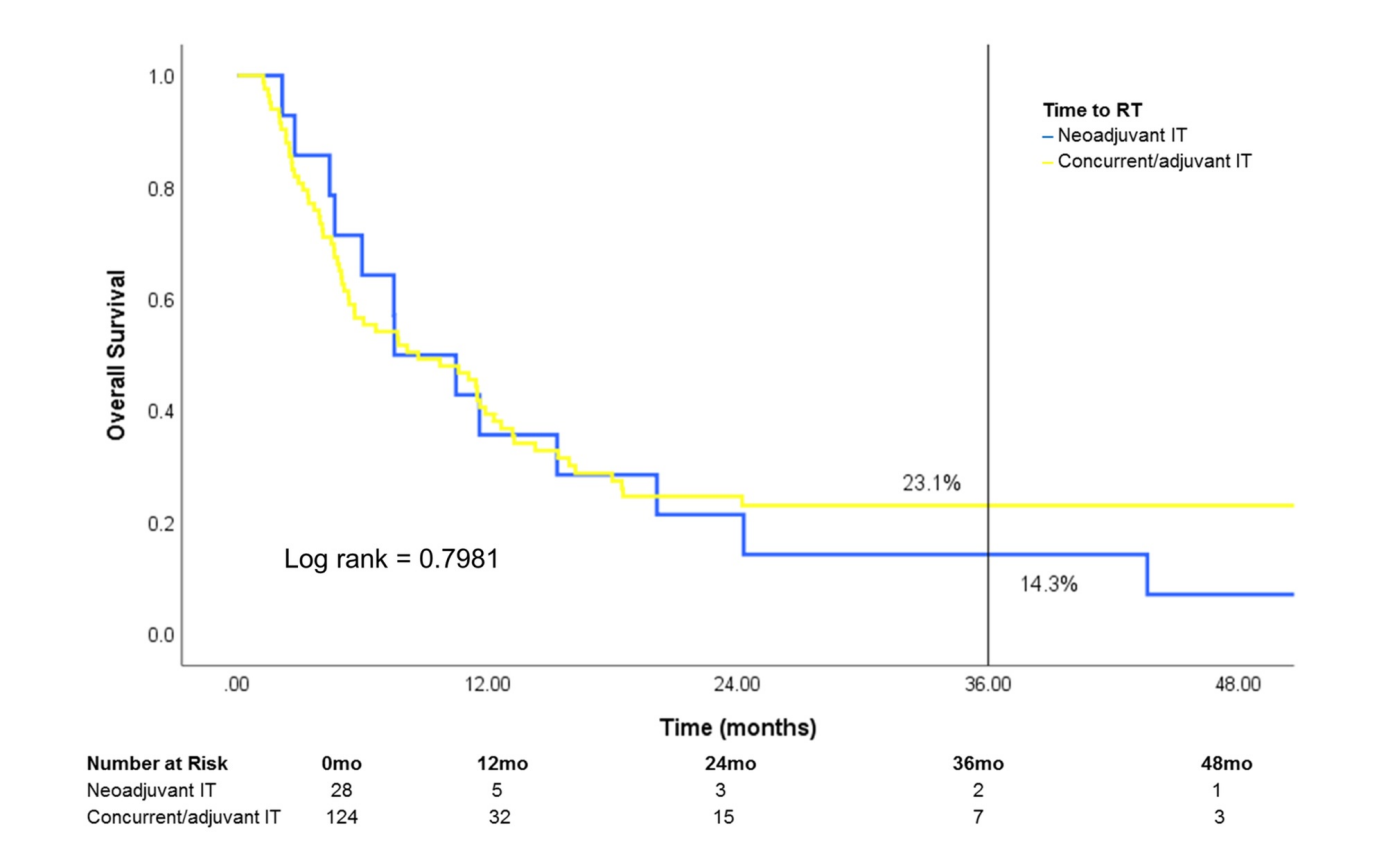
Effect of IT timing on overall survival in patients who received RT to the bone Patients who received RT to the bone and IT (n=152) were stratified by IT timing RT: radiotherapy; IT: immunotherapy

Of patients treated with both RT and IT, survival was similar regardless of IT timing [neoadjuvant IT (IT >30 days prior to RT), concurrent IT (IT within 30 days of RT) or adjuvant IT (IT >30 days after RT)] (Figure 8). After stratification by site treated, patients who received treatment to the brain showed a difference in survival that was marginally significant in favor of concurrent/adjuvant IT (log rank: 0.0749) (Figure 9). Conversely, survival was similar in patients who received treatment to STV sites or bone when stratified by IT timing (Figures 10, 11).

## Discussion

For patients receiving IT for metastatic melanoma, RT treatment with HFRT was associated with superior survival compared to CFRT. Moreover, patients treated with HFRT was associated with significantly greater survival benefit from the use of IT compared to those treated with CFRT, especially patients treated with RT to the brain and STV sites. We obtained similar findings after PSM analysis, including matching by irradiated sites.

Currently, there is no evidence to guide the optimal dose-fractionation of RT in combination with IT for the treatment of any malignancy. There are only a handful of ongoing clinical trials evaluating RT fraction size in combination with IT [17]. However, available clinical evidence has shown that RT with higher dose-per-fraction, when combined with IT, results in favorable outcomes in metastatic melanoma. Several studies have reported evidence of the abscopal effect with stereotactic body RT (SBRT) in metastatic melanoma patients receiving IT [18-20]. Moreover, in a recent systemic review of studies reporting the therapeutic efficacy of combined RT and ipilimumab in metastatic melanoma, patients who received higher doses-per-fraction (>3 Gy) showed better clinical outcomes [21]. Similarly, preclinical evidence skews toward HFRT/SBRT fraction sizes to be more effective in providing therapeutic synergy with IT [17].

In this study, we showed that metastatic melanoma patients who received IT had significantly higher OS if they received HFRT (>5 Gy/fraction) rather than CFRT (≤5 Gy/fraction). Moreover, we further ascertained the survival contribution of IT in patients receiving palliative RT by comparing patients treated with HFRT or CFRT, with or without IT. Although patients receiving either CFRT or HFRT were associated with significant improvement in three-year OS when IT was also given compared to their counterparts who did not receive IT, the absolute survival benefit from IT in patients receiving HFRT was greater than those receiving CFRT. As such, our analyses suggest that there is a differential effect with different RT dose-fractionations, with the combination of HFRT and IT potentially exhibiting greater therapeutic effect that translates into improved survival. One possible reason for this observation of ours is that patients who were treated with CFRT for palliation may potentially benefit less from IT. Greater number of RT fractions and a generally larger area of treatment with CFRT may lead to more substantial lymphopenia, which can hinder tumor cell eradication by cytotoxic T lymphocytes [22-24]. Another possibility is that patients receiving HFRT may benefit more from IT, perhaps due to the synergistic effects of higher dose-per-fraction with checkpoint inhibition, as previously discussed [17-20].

The treatment site is likely an important determinant of the response to therapy. It has been suggested that RT to lungs and liver is more immunogenic than to other organs [25,26]. Furthermore, although brain parenchyma was previously considered to be immune-privileged, recent evidence has shown that immune cells can cross the blood-brain barrier, and brain metastases from melanoma respond to immune checkpoint inhibitors [27-29]. Consistent with this, our analysis demonstrated that patients with metastatic brain or STV lesions demonstrated a greater survival benefit with the addition of IT to HFRT compared to those with osseous metastatic lesions.

There are several limitations to this study. First, differential misclassification may still exist toward HFRT being a more effective treatment than CFRT against metastatic melanoma using the NCDB database. For example, patients treated with radiosurgery may have had an intracranial disease of lower volume than those who received whole-brain RT; similarly, patients treated with CFRT to the bones were more likely to harbor diffuse osseous metastases than those who underwent SBRT. Additionally, CFRT may be less effective in eradicating melanoma cells, which are less radiosensitive with a lower α/β ratio [30]. Finally, HFRT dosing can often achieve a higher biologically effective dose to the tumor lesions and result in greater tumoricidal effect. To circumvent these biases, we minimized the potential for misclassification by comparing HFRT or CFRT patients who received IT with their respective counterparts who did not. As such, we were able to determine the benefit derived from IT in HFRT or CFRT patients separately, rather than directly comparing HFRT patients with CFRT patients. Second, this is a retrospective study that utilized data collected only from facilities accredited by the CoC, which may generally include patients who received higher quality care and had better outcomes. Third, while we adjusted our models for important covariates, we were unable to account for factors that were not available in the database, including total disease burden, size and number of lesions treated by RT, or the type of immunotherapeutic agents utilized. Finally, we were unable to exclude the possibility that patients may have received multiple courses of RT to the same or different disease sites.

Despite these limitations, our study has several strengths. In addition to defining the two dose-fractionation criteria by a commonly-used threshold of 5 Gy/fraction, we only included patients who received clinically meaningful doses of RT at any given dose-per-fraction to ensure that we captured the appropriate cohort for our analysis. Furthermore, we examined the survival differences imparted by IT by including HFRT-/CFRT-alone groups in our four-arm analysis. Additionally, our results after PSM were very similar to that of the original analysis, indicating that selection bias had a minimal effect on our analysis.

## Conclusions

Our analysis demonstrated that patients treated with IT in combination with higher dose-per-fraction using HFRT had superior OS compared to those treated with IT and CFRT. Furthermore, we observed a differential effect of IT on RT fractionation, with significantly greater improvement in OS when IT was utilized in patients treated with HFRT compared to CFRT, especially in patients who received RT to the brain and STV sites. Altogether, we believe our study contributes to the literature by addressing a critical gap of knowledge regarding the optimal dose-fractionation of palliative RT for metastatic melanoma in conjunction with IT, thereby underscoring the significance of and providing potential guidance for future prospective studies addressing this issue. Furthermore, designing trials by utilizing specific immunotherapeutic agents is essential to better evaluate the interactive effects with RT.
